# Estimating acid soil effects on selected cereal crop productivities in Ethiopia: Comparing economic cost-effectiveness of lime and fertilizer applications

**DOI:** 10.1371/journal.pone.0280230

**Published:** 2023-01-12

**Authors:** James M. Warner, Michael L. Mann, Jordan Chamberlin, Chilot Y. Tizale

**Affiliations:** 1 International Food Policy Research Institute, ILRI Campus, Addis Ababa, Ethiopia; 2 Department of Geography, The George Washington University, Washington, DC, United States of America; 3 Food and Agriculture Organization of the United Nations, Rome, Italy; 4 Ethiopian Institute of Agricultural Research, Addis Ababa, Ethiopia; The Ohio State University, UNITED STATES

## Abstract

Acid soils are a major constraint to agricultural productivity in many parts of sub-Saharan Africa, including Ethiopia. Restoring soil pH to optimal ranges for agriculture can have a significant impact on yields, particularly for acid intolerant crops like wheat and barley. The application of agricultural lime is the standard corrective, although the large application requirements, lack of farmer awareness, and weak or non-existent lime supply chains make this a complex problem to address at scale. To date, no large-scale farmer trials of lime application have been undertaken in Ethiopia. This leaves open the question to local policy makers as to the economic benefits given the enormous capital and logistics investments required. To help address this we leverage existing spatial edaphic data and longitudinal crop surveys to simulate the productivity impact of varying lime and fertilizer applications. Our estimates find the impact of moving pH from 5.5 to 6.5, modeled as a lime soil remediation strategy, increases yields by 22% and 19% for wheat and barley, respectively. In addition, at lower pH levels our models indicate that commonly used nitrogen-based fertilizers are less cost-effective. For wheat in highly acidic soils, we find that fertilizers cost over two times as much as a single application of lime over a five-year period. The cost savings of the use of lime reaches as high as 121% of average one-year agricultural household income for wheat; with barley these savings are lower but still substantial at 24%. In general, we advocate for an integrated soil fertility management strategy that applies appropriate levels of fertilizer on pH balanced soil. If successful, Ethiopia’s acid soil reclamation could become a modest version of Brazil’s successful “cerrado miracle” and serve as an example for Africa.

## 1. Introduction

Over decades of intensive utilization, small-scale farmers in Africa have removed large quantities of nutrients from their soils without sufficient inputs to replenish them [1, 2]. Even though Ethiopia holds a substantial proportion of East Africa’s best croplands, the natural characteristics, intensive use of fertilizers on many of Ethiopia’s lands have led to extensive soil acidification and abandonment. This, coupled with the loss of topsoil, creates a challenging environment for the country to increase staple crop yields and food supply. While there exist some empirical assessments of the extent of soil acidity in Ethiopia, national estimates of impacts on yield gaps have not been carried out [3]. As such, there have been no studies addressing how to close those gaps in a cost-effective manner at a national scale.

Soil acidification is a major cause of declining soil fertility in many parts of the humid sub-tropics, particularly in tropical Oxisols and Urtisols soils. Soils become acidic when basic elements such as calcium, magnesium, sodium and potassium held by soil colloids are replaced by hydrogen ions. This removal of bases can occur through natural processes, such as leaching caused by rainfall, or anthropogenic processes, such as intensive use of ammonium-based fertilizers and continuous cropping without organic inputs [2, 4]. The impact of N-based fertilizers is case dependent but can increase acidity if some N is lost to leaching [4]. One research group suggests that for every 1 kg of urea applied, nearly 1.8 kg of calcium carbonate is required to neutralize the treated soil [5]. Moreover, anthropogenically induced acidification is likely a major contributing factor to farmland abandonment in Ethiopia’s more marginal croplands [6].

Acidification has a number of direct and indirect effects on plant health and development. Correcting soil acidity through the use of lime can serve as a foundation of a good soil fertility program. Maintaining proper soil pH has a variety of primary and secondary benefits including critically, increasing yields and the stimulation of microbial activity. In some experimental wheat plots, lime application can increase root length density by >100% and yields by up to 200% [7]. Most varieties of wheat and barley grow best in soils with a pH ranging from 6 to 7, with 6.5 often mentioned as the target level [8–10].

Areas of high acidity are located throughout the western Ethiopian highlands, but particularly in the western and central highlands of Oromia and Amhara [11–13], and highland areas of SNNP [14]. In all it is estimated that 40% of Ethiopia’s arable lands are acidic [11]. More recent assessments have indicated that soil acidity is expanding in scope and magnitude in Ethiopia, seriously affecting crop production [15]. For example, in some barley, wheat and fava bean growing areas of central and southern Ethiopian highlands, farmers have shifted to producing oats that are more tolerant to soil acidity [16, 17]. Many farmers practice a barley–fallow–oats rotation system to reduce the negative effects of soil acidity and improve soil fertility [18]. However, this rotation system is not sustainable [19], and addressing soil acidity with lime seems critical for longer term sustainability.

Lime is often referred to as the foundation or “workhorse” in acid soils [20], and a multitude of researchers have shown the effect of lime on soil pH. Studies also indicate that joint application of lime and fertilizer (components of an integrated soil fertility management strategy) have significant synergistic effects on grain yields. Caires et al. [7] evaluated the effects of wheat yield and root growth and found that wheat yield was significantly increased by lime combined with fertilizer. Research indicates that lime applications have multi-year impacts which conservatively last between 5 to 7 years. However, a lime application benefits can extend to as many as 10 to 12 years from a single lime application, implying the impacts of lime application compound over several years [21–23].

Field studies inside of Ethiopia and other developing nations have found consistent benefits of lime application, including the expected reduction of acidity [24, 25]. These experiments have found a 90–130%+ increase in yields on treatment plots [24, 26], increased nutrient availability [25, 27], and indications of sustainable yields for soy-wheat rotation, and malt barley production in acidic soils using lime in conjunction with N, P and K (NPK) [27]. Overall, while liming application amounts, crop type, and productivity responses vary widely, overwhelming evidence suggests significant productivity responses with lime applications in both international and Ethiopian studies. It is important to note that most of these studies are done at the test plot level and, to the best of our knowledge, no larger pilot programs have evaluated the effects of lime application of representative farmers in Ethiopia, or Africa more generally.

In this article we aim to partially address the information gap created by the lack of large-scale national lime trial research. We do this by leveraging statistical methods and exploiting the natural spatial variability in pH and existing longitudinal crop productivity data. We focus on wheat and barley because they are among the most important cereal crops grown in Ethiopia’s highlands. Given the resource constraints of agricultural interventions in Ethiopia specifically, but Africa more generally, we compare the cost effectiveness of commonly used fertilization methods with the potential impacts of soil remediation through the application of lime. Specifically, we evaluate the quantity and cost of annual fertilizer applications required to obtain the same productivity gains from a single lime application over a five-year period. This comparison is not to argue for an “either or decision” e.g., choosing between lime and fertilizer, but critically, to explore whether or not lime should be considered an important and cost-effective intervention in Ethiopia. As an important caveat, it is critical to acknowledge that our analysis uses secondary data projected over the sub-kebele area and is not at the individual plot level. While this level of aggregation depicts general impacts of pH and crop productivity, we believe the potential impacts of lime and pH balanced soil on crop productivity would be greater at the plot level. We cannot directly assess the application of lime as a corrective because it is largely unavailable to farmers. Therefore, we estimate the marginal effects of lime exploiting the spatial variability of soil pH and their resultant differences in reported crop yields, as an imperfect, yet meaningful, proxy.

## 2. Materials and methods

In this analysis, we leverage statistical methods and spatial variability in pH, coupled with longitudinal crop-cut data, to model the yield responses of wheat and barley. Our basic strategy is to use empirical insights about these crop-specific yield-pH relationships, in concert with reasoned assumptions about soil pH responses to lime, as the agronomic basis of a location-specific cost comparison of two alternative interventions, lime and fertilizer. We explore both the extent to which–and where–farmers would benefit from the application of lime to increase yields in comparison to yield gains attainable through nitrogen (N) fertilizers. Specifically, this analysis provides a comparison of the present value or “current cost” of lime to that of fertilizer required to provide the same yield gain over a five year-period. Present value is the current value of a future flow of money discounted to take into account for compounding at some specified rate of return (discount rate). In this case it is the “current cost” of future payments for lime or fertilizer application(s), or the amount you would need to hold aside today to make these purchases.

### a) Data & study area

#### Agricultural sample survey data (2010–2016)

Our analysis uses annual agricultural production data from the Central Statistical Agency’s (CSA) Agricultural Sample Survey (AgSS) between 2010 and 2016 [28]. For this study we utilize measures of crop yields in metric tons per hectare (mt/ha), the percentage of plots applied with improved seeds, the percentage of plots that have access to irrigation, the percentage of plots actively receiving extension agent support, the intensity of chemical fertilizer use (kg/ha), and the percentage crops reported as damaged by the farmer. Damages include several factors including weather-related, pests, etc. All AgSS data is reported at the sub-kebele level, which is the unit of analysis for the remainder of this paper. A sub-kebele, or village, is below the smallest government administrative area (kebele) and is representative of approximately 200 agricultural households. For more details about the AgSS refer to S1 File—Appendix A.

#### Soil properties

**Fig 1:**
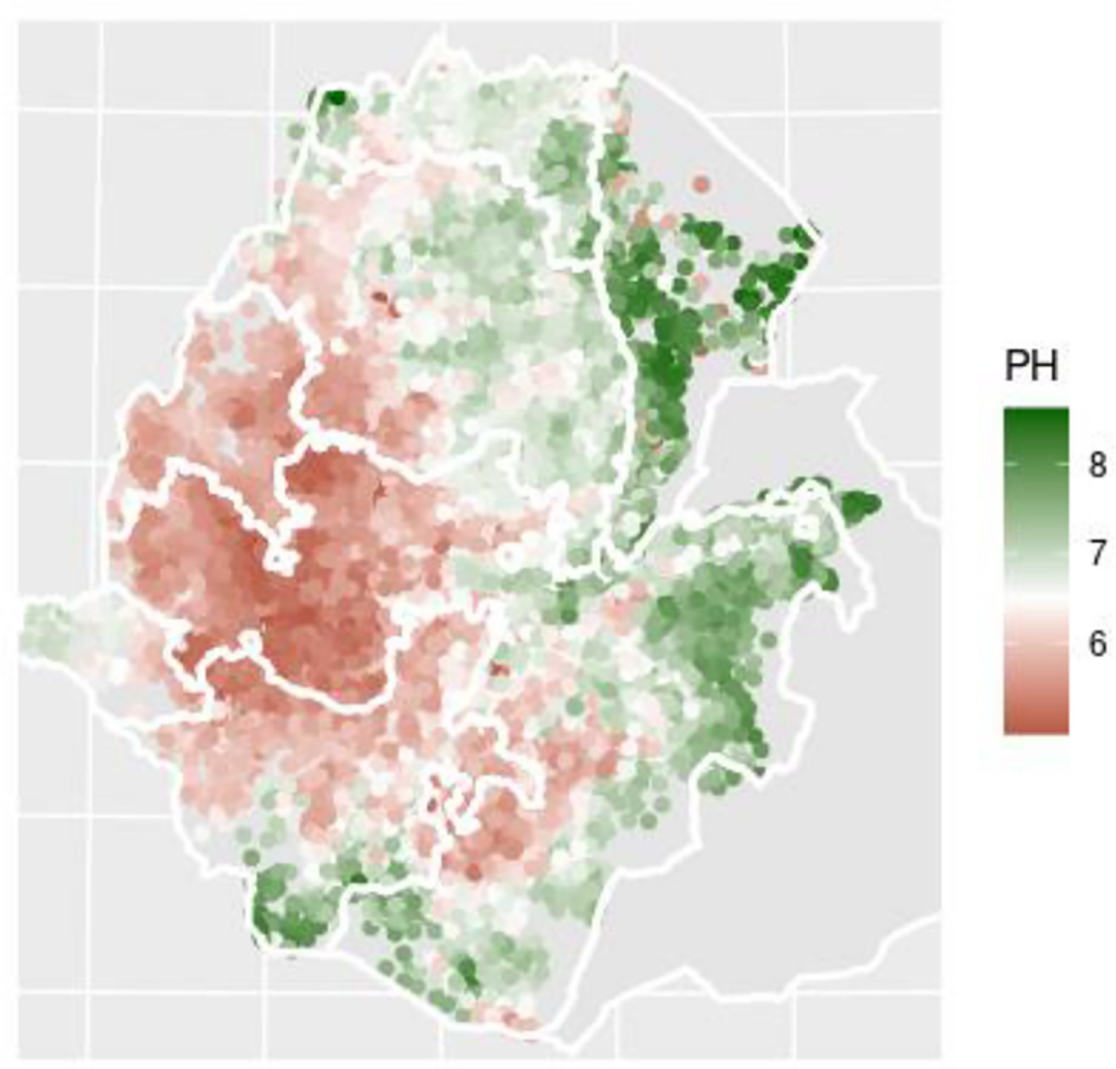
Ethiopia’s soil pH balance. Contains information from OpenStreetMap and OpenStreetMap Foundation, which is made available under the Open Database License.

Data on soil properties were collected from the Ethiopian Soil Information System (EthioSIS). EthioSIS was charged with gathering soil samples from all major growing regions to help provide detailed information on soil fertility, and to provide localized fertilizer recommendations [29]. For this study we access sub-kebele estimates of EthioSIS measures like soil pH, as well as measures of cation exchange capacity, soil organic content, and sand content at approximately 15cm depth. Cation exchange capacity is a measure of a soils ability to hold and supply nutrients to plants and is affected by soil pH. We focus on two key categories of soil acidity, moderately acidic soils (5.5 < pH ≤ 6) and highly acidic soils (≤ 5.5). Soil properties are aggregated to the sub-kebele level. For more details on EthioSIS see S1 File—Appendix A. Soil pH levels measured by EthioSIS can be seen below in Fig 1.

#### Remotely sensed variables

This study controls for the impacts of drought and elevation with remotely sensed data. We obtain hydrological variables from the University of Idaho’s TerraClimate data [30] and utilize a measure of drought called the Palmer Drought Severity Index (PDSI) [31]. PDSI is an effective measure of longer-term drought. For more details on TerraClimate see S1 File—Appendix A. Elevation data is obtained from NASA’s Shuttle Radar Topography Mission (SRTM) at 90-meter resolution [32].

#### Other variables

Two dummy variables were used to capture areas impacted by national agricultural policy interventions which receive targeted external support. These two programs include the Agricultural Growth Program (AGP) for high potential growth areas, and those designated for social protection programs using the Poverty Safety Net Program (PSNP). We also control for the major agroecological zones of Ethiopia with data from The Water Land Resource Center [33] Additionally, access to markets is controlled for using Euclidean distance to the nearest populated town with more than 50,000 people [34]. This paper was generated in R, stargazer, R-markdown, knitr, and ggplot2 with [35–41].

### b) Soil pH responses to limestone applications

For operational purposes, it is important to point out that pH measures only a portion of a soil’s acidity and should not be exclusively used to determine lime requirements. To better measure needed lime requirements the soils “buffer capacity” is projected as a function of sub-kebele values of CEC as well as base saturation estimates values determined by a pH-base saturation model [9].

To estimate lime application rates needed to raise pH levels to the desired levels, there are at least seven items to consider in the Ethiopian context [10], shown in Eq 1:

$$L=(CEC)\left(\frac{BS_{t}-BS_{o}}{2(1-BS_{o})}\right)\left(\frac{TD}{6}\right)(CCE)(F)$$ (1)

Where *L* is the lime required (mt/ac) can be determined by: cation exchange capacity (CEC), *BS*_*t*_ and *BS*_*o*_ are the target and original base saturation rates at the current and at the desired pH, respectively, tillage depth (TD) in inches, calcium carbonate equivalent (CCE) of the chosen lime material, relative fineness of the liming materials (F), and adapting estimates to Ethiopian conditions.

Details on the use of this formula for estimating the amount of lime required to obtain a pH of 6.5 are outlined in section E of S1 File—Appendix A.

### c) Estimating yield responses to pH changes

#### Exploring bivariate relationships

Bivariate plots are used to help frame the discussion of the relationship between yields and fertilizer or lime applications. We use sub-kebele level observations from the AgSS, pooling data from the 2010–2016 growing seasons. Because there are thousands of observations, they are summarized with Loess smothers. In each figure the colored lines are the result of Loess smothers tracing the non-linear relationship between the observations of the x and y factors, and the 95% confidence intervals are in gray. These plots, while informative, are simplistic as the impacts of any variable should be evaluated in a multivariate context.

#### Multivariate estimation strategy

In order to examine the marginal effect of grain output per hectare to changes in soil acidity, we control for the effects of other variables such as water availability, soil properties, elevation, policy interventions, and management differences. To do this a regression is used on all sub-kebeles for 2010–2016 with year and agricultural zone fixed effects. This regression allows us to examine how a one unit increase in fertilizer or pH relates to wheat or barley yields while controlling for other potential determinants of productivity. To better address the highly non-linear nature of many of these relationships, polynomials are applied to all independent variables. The order of the polynomial is established using recursive nested ANOVAs to test the hypothesis that a model *M*_1_ is sufficient to explain the data against the hypothesis that a more complex model *M*_2_ is required. In our yield response model, we control for a variety of independent variables. These are defined Table 1 below:

**Table 1:** Variable definitions and sources.

Name	Definition	Source
PH	Mean pH	EthioSIS
CEC	Cation exchange capacity	
SOC	Soil organic content (%)	
SND	Sand content (%)	
Elevation	Elevation (m)	Remotely Sensed
PDSI	Palmer drought severity index ([–5,5])	
Yield	Observed crop yield in (mt/ha)	AgSS
Ext.Area	Area advised by extension agents (%)	
Imp.Seed	Area applied with improved seeds (%)	
Irg.Area	Area irrigated (%)	
Damage	Area reported as damaged by farmer (%)	
Fert	Chemical fertilizer intensity (kg/ha)	
DistPP	Distance to populated place	Other
PSNP	PSNP dummy variable	
AGP	AGP dummy variable	
Year	Year of harvest	
Agri.Eco.	Agro-Ecological Area	

In order to test the sensitivity of these regressions a number of alternative specifications are presented in S1 File—Appendix B.

### d) Spatially varying input prices

A key part of our approach is recognizing that, like agronomic response, the economic returns to lime application differs by location. This is particularly salient in a country like Ethiopia, where complex terrain and market access have important implications for marketed prices and, consequently, the economic attractiveness of productive investments [42]. In our analysis, we address this through spatially explicit estimates of agricultural lime application cost.

Because the market for agricultural lime has not yet been developed in Ethiopia, market prices are not available. We therefore estimate the distribution costs of delivered lime to cooperative unions or other private input distributors on the basis of current crusher locations at Butajira, Guder and Dejen. One metric ton (mt) of lime at each crusher location was assumed to cost 750 Ethiopian Birr (ETB). To conform to our period of analysis, we use December 2016 exchange rates ($1 = 22.07 ETB). From each crusher location, the cumulative travel time from all locations in the country was calculated on the basis of a spatial cost-distance model, which factored in road network data [43], discounted by terrain steepness. This price includes transportation costs from lime crushing facilities to distribution centers but does not include either the “last-mile” transport or application costs. We assume a fixed loading cost of 100 ETB/mt and variable transport costs of 60 ETB/mt/hr. Resulting estimates of delivered lime costs are shown in Fig A2 in S1 File—Appendix A, with representative lime cost estimates provided for major towns of interest listed in Table 2 below.

**Table 2:** Input distributors lime cost predictions for different locations.

City	Nearest Crusher	Travel Time	Distributors Costs
		(hr)	(ETB/mt)
Adigrat	Dejen	16.2	1,821
Axum	Dejen	14.0	1,689
Gonder	Dejen	8.9	1,381
Bahir Dar	Dejen	6.0	1,213
Dessie	Butajira	8.6	1,367
Debre Markos	Dejen	1.7	954
Debre Birhan	Butajira	4.5	1,121

### e) Cost comparisons of lime and fertilizer application

To understand differences between the cost of lime and fertilizer we need some normalized basis of comparison. To do this we estimate the marginal response in yields, corresponding to differences in pH and fertilizer for wheat and barley. We then estimate, for the average sub-kebele hectare, how much it would cost to reduce acidity from, for instance, a pH of 5.5 to 6.5 through the application of lime, and the average yield response of this intervention measured in mt/ha. In contrast, we can also estimate the amount and cost of fertilizer required to obtain the same yield response (mt/ha) expected from that lime treatment and compare them. This comparison is presented throughout the paper as ‘X Cost of Lime,’ and is the economic costs of fertilizer divided by the cost of lime—for some specified yield response—typically that of bringing the observed pH up to 6.5.

### f) Present value of future lime and fertilizer expenditures

To compare the costs of lime and fertilizer, it is critical to note that fertilizers require annual applications, while lime is assumed to be applied once every five years. Fertilizer applications therefore need to be thought of as a repeated annual cost over a five period, subject to a present value calculation.

For present value calculations we assume that the annual costs of Urea and NPS fertilizer are 11.5 ETB/kg or 0.52, reflecting costs during our period of analysis (Dec. 2016). These costs are discounted across five years, at an annual discount rate of 8% and an inflation rate of 13.4%. These rates reflect savings interest rates and consumer price indices during our time period of interest [44, 45].

## 3. Results and discussion

### a) Descriptive analysis

#### Bivariate plots

**Fig 2:**
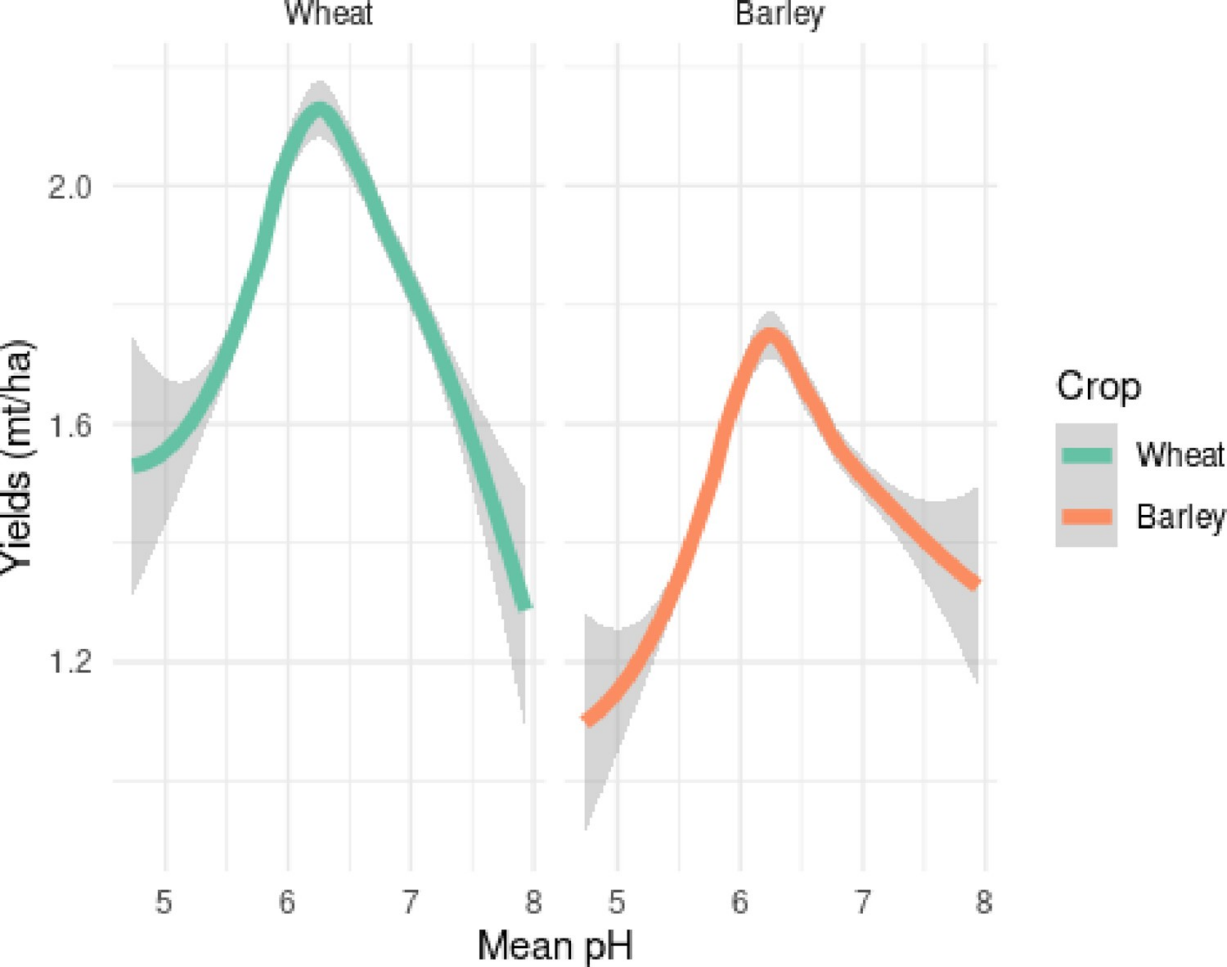
Bivariate relationship between crops yields and soil pH.

To test whether our data conform to the established relationship between pH and wheat and barley yields, we explore the relative yields and pH, at the sub-kebele level. Fig 2 graphically depicts this correlation.

**Fig 3:**
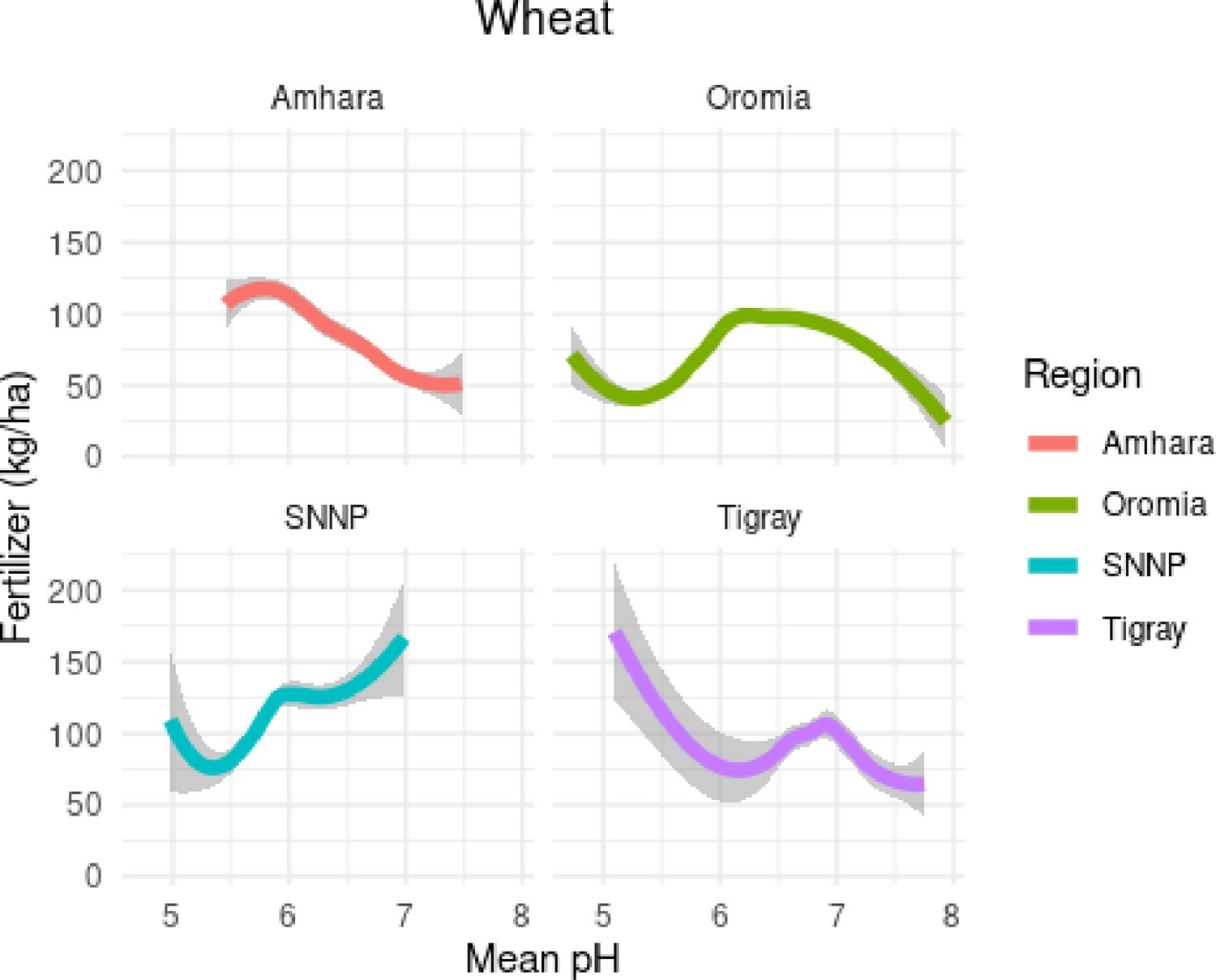
Estimated relationship between fertilizer use and soil pH for wheat.

Consistent with literature, we see a concave response of yield to pH for wheat and barley, both peaking near 6.5. However, yields, are impacted by a number of edaphic and management variables. To test if wheat farmers are adapting to highly acidic soils by applying more fertilizer, we plot observations of intensity of fertilizer use (kg/ha) against soil pH for wheat in Fig 3.

Fig 3 depicts regional differences in fertilizer application and pH levels. In Amhara, lower pH values correspond to significantly higher utilization of fertilizer in wheat (p < 0.01), implying that for each one unit decrease in pH (more acidic) farmers apply an additional 45.8 Kg/ha of chemical fertilizers. In Tigray, there are a handful of sub-kebeles applying large amounts of fertilizer to highly acidic soils, and across the region fertilizer application decrease with pH (p < 0.05). Meanwhile, farmers in Oromia appear to target additional fertilizers to areas with ideal pH, and SNNP appears to significantly increase fertilizer applications in soils with higher alkalinity (p < 0.01).

This variety of regional responses to pH likely reflects local policy and extension approaches to address lower than expected yields. Anecdotal evidence from a former Amhara official suggests that fertilizer was heavily emphasized over balancing soil pH to boost flagging yields. Although suggestive, the effectiveness of these mitigation strategies will depend on the relative costs of annual fertilizer applications, and the frequency of lime application. Given this finding, farmers utilizing greater than average amounts of fertilizer, especially urea, may now be suffering from “induced acidification” especially in Amhara and Tigray. Induced acidification occurs during the growth period when crops absorb basic elements such as Ca, Mg, and K to satisfy their nutritional requirements, and removing these lime-like nutrients [17].

#### Moving pH from 5.5 to 6.5 –a simplified case

Based on expert opinion from soil scientists knowledgeable about Ethiopian soil conditions, we can estimate the cost of lime to move the pH one unit, *from 5*.*5 to 6*.*5*, with approximately 3.12 metric tons of lime per hectare. The amount of lime required is approximated using the trend line in Fig A1 in S1 File—Appendix A. We also assume the average distributed price for the major growing regions (Amhara, Oromia, SNNP, and Tigray) of lime is 1,681 ETB/mt or $76. This average price includes transportation costs from lime crushing facilities to distribution centers but does not include either the “last-mile” transport or farmer application costs. Given these assumptions, the average cost of increasing the pH one unit is estimated to be approximately 5,266 birr or $239.

### b) Estimating multivariate response of wheat yield to pH changes

Estimation results from the model described in the “Multivariate Estimation Strategy” section are shown below (Table 3). This regression allows us to examine how a one unit increase in fertilizer or pH effects wheat and barley yields while controlling for other potential determinants of productivity. The results largely match our expectations and coefficients are statistically significant at higher orders (indicating the statistical importance of non-linear estimation). A variety of alternative specifications are presented in S1 File—Appendix B.

**Table 3:** Wheat & barley yield regression estimates.

	Wheat Yield		Barley Yield	
	Model 1	Model 2	Model 3	Model 4
p(PH, 1)	69.174***	69.146***	86.173***	87.768***
p(PH, 2)	-61.306***	-61.300***	-20.678**	-20.826**
p(PH, 3)	-33.008***	-33.000***	-23.731***	-23.768***
p(PH, 4)	23.537**	23.545**		
p(Fert, 1)	100.841***	100.843***	78.689***	78.414***
p(Fert, 2)	-5.796	-5.801	-30.988***	-30.960***
p(CEC,1)	-57.452***	-57.444***	2.685	
p(CEC,2)	-13.71	-13.709		
p(CEC,3)	38.134***	38.128***		
p(SOC, 1)	34.309**	34.292**	78.092***	78.074***
p(SOC, 2)	-21.697**	-21.688**	-28.931***	-28.409***
p(SND, 1)	-63.920***	-63.910***	-4.343	-5.662
p(SND, 2)	-29.640***	-29.628***	-48.862***	-48.979***
p(SND, 3)			11.864	11.849
p(SND, 4)			32.812***	32.825***
PDSI	-29.124**	-29.126**	-8.835	-8.721
Elevation	103.721***	103.724***	141.295***	141.200***
p(Ext.Area, 2)	51.308***	51.306***	20.405**	20.410**
p(Ext.Area, 2)			12.467*	12.534*
p(Imp.Seed,1)	24.051**	24.050**	5.747	5.756
p(Imp.Seed,2)	-43.741***	-43.746***		
p(Imp.Seed,3)	29.173***	29.179***		
Irg.Area	-0.339		-3.617	
p(Damage, 1)	-152.643***	-152.644***	-120.112***	-119.982***
p(Damage, 2)	37.123***	37.114***	29.418***	29.239***
p(Damage, 3)	23.274***	23.274***	15.329**	15.360**
p(Damage, 4)	28.914***	28.926***	19.686***	19.641^{***
DistPP	-31.793***	-31.798***	0.847	
Constant	16.709***	16.708***	12.960***	12.949***
Fixed effects	Yes	Yes	Yes	Yes
Adj. R-squared	0.225	0.225	0.226	0.226
Residual Std. Error	8.532 (df = 6965)	8.531 (df = 6966)	7.130 (df = 5908)	7.128 (df = 5911)

***p < .01

** p < .05

* p < .1

To isolate the marginal effect of pH and fertilizer on yields, we hold all other variables at their mean (fixed effects variables are set to a specific year and zone), while letting the variable of interest vary. As expected, highly acidic soils (low pH) show low levels of productivity, increasing significantly with higher pH, until an inflection point. Response curves from models 1 and 3 are shown below. Because lime is largely unavailable to most farmers, these curves are based on spatial variability in observed pH and yields, rather than on plot-level applications of lime. As such, they are indicative of the impact of lime, and are not definitive results. As such, our spatially aggregated results likely underestimate relative responses at the individual household level. Further research, at the plot level of the representative farmer using lime, is strongly recommended.

**Fig 4:**
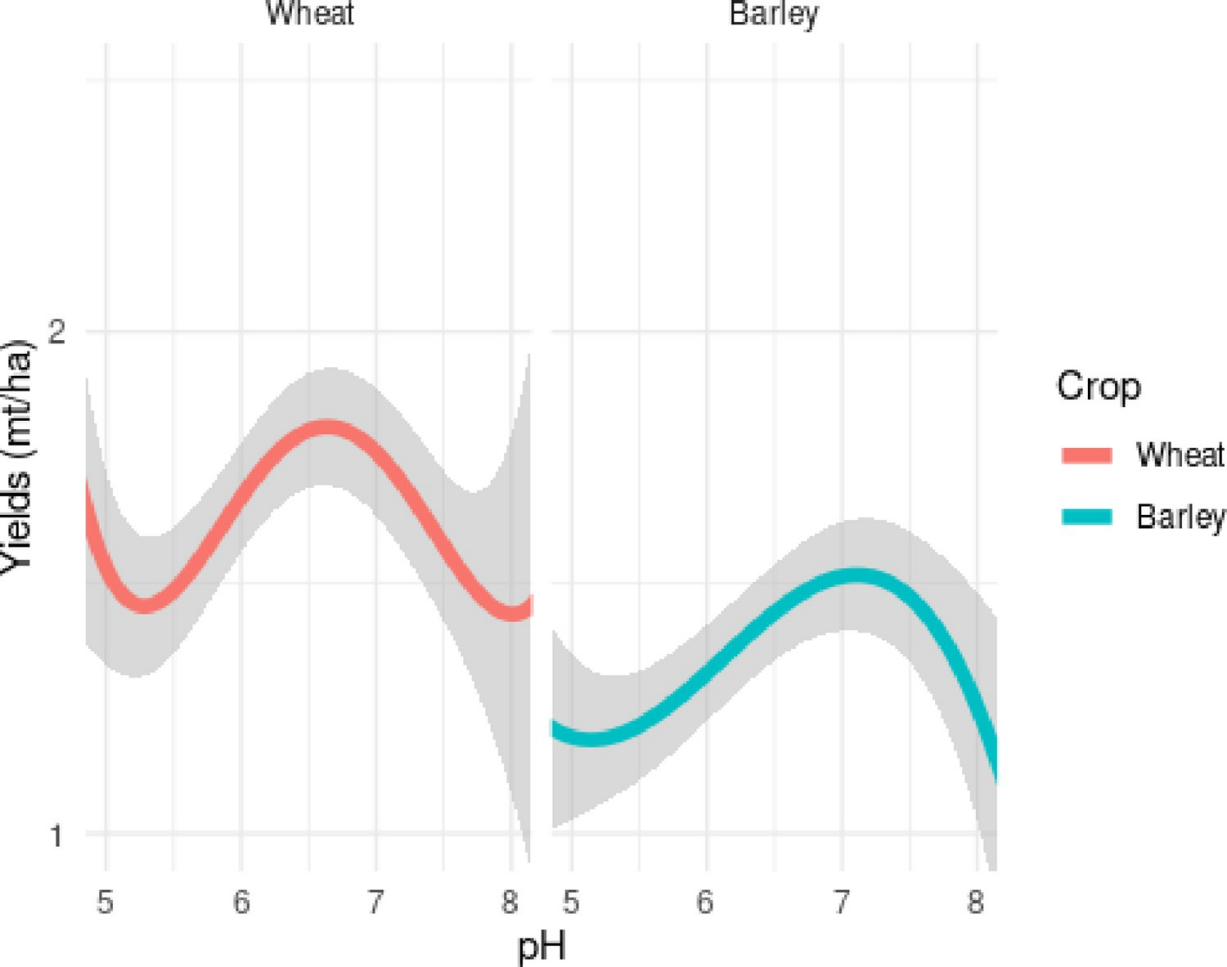
Estimated response of wheat yield to changes in pH.

To understand the response of yield to changes in pH in a multivariate context, controlling for weather management and edaphic properties, we plot the estimated response curve from models 1 and 3 in Fig 4.

Polynomial estimates in Fig 4 are used to estimate the yield response obtained from moving pH to a minimum of 6.5. In line with literature, we see a convex shape peaking near a pH of 6.5–7 for both wheat and barley. However, there are a small number of observations at the tails of the pH distribution where yields increase. These observations are infrequent, as reflected in the increasing 95% confidence intervals, but likely reflect the extraordinary application of fertilizer in some low productivity areas, especially in Tigray and Amhara (see Fig 3).

The yield gains obtained from the generalized case of moving soil pH from 5.5 to 6.5 for both crops are presented in Table 4 below. We see differences between soils with a pH of 5.5 to those with 6.5 corresponds to a 21.5% increase in yields for wheat, and 18.5% for barley, ceteris paribus. It is also notable that our estimated yield increases are significantly lower than those reported in controlled trials [24, 26]. The differences between our methods and theirs are numerable. First and foremost, our methodology only simulates a change in pH from 5.5 to 6.5 based on the natural variability of yields across the country, and controls for a limited set of climatic and management practices. Our yield estimates are likely understated because none of the additional benefits obtained from lime application can be simulated—like the introduction of micro-nutrients and reduction in mineral toxicity. Additionally, controlled trials reflect ideal conditions and modern management practices like soy rotation, and therefore often overstate real-world yield gains [46, 47].

**Table 4:** Estimated yield gains from changing pH from 5.5 to 6.5.

Crop	Yield Gain	Yield Gain
	(mt/ha)	(%)
Wheat	0.3	21.5
Barley	0.2	18.5

We can also look at the spatial distribution of productivity gains based on actual pH levels. S1 File—Appendix B presents maps (Figs B1-B2 in S1 File) showing the impact of increasing the observed pH to a minimum of 6.5 for both barley and wheat.

**Fig 5:**
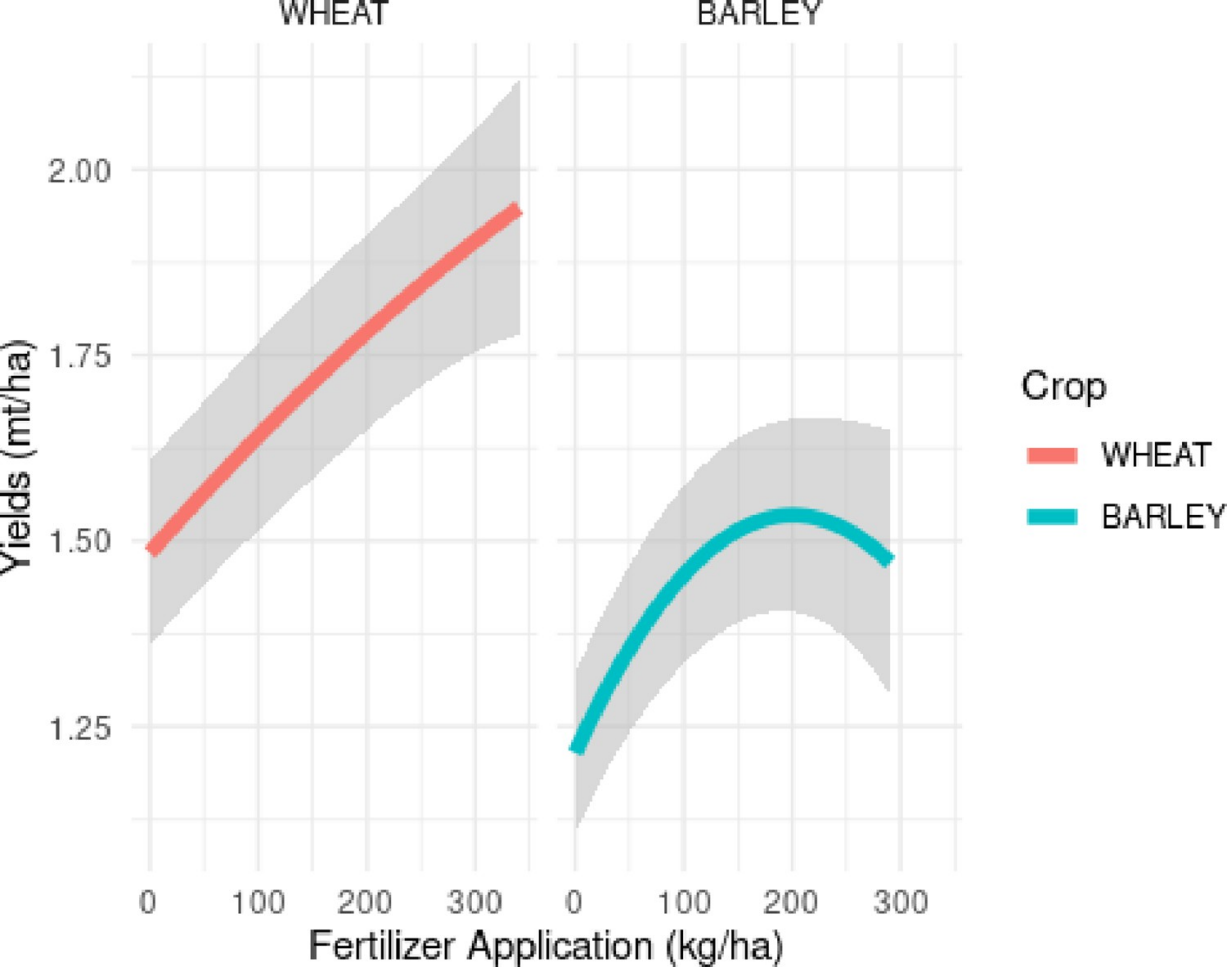
Marginal yield response of wheat yield to changes in fertilizer application holding pH at 5.5.

To evaluate the marginal response of yields to fertilizer applications, we plot the estimated curves from models 1 and 3 in Fig 5 below:

Fig 5 is used to estimate the yield response from the application of fertilizer. For wheat and barley, adding an additional 100 kg per ha of fertilizer, holding pH at 5.5, corresponds to a 0.16 and 0.24 mt/ha increase in yields.

### c) Targeting interventions given current conditions

**Fig 6:**
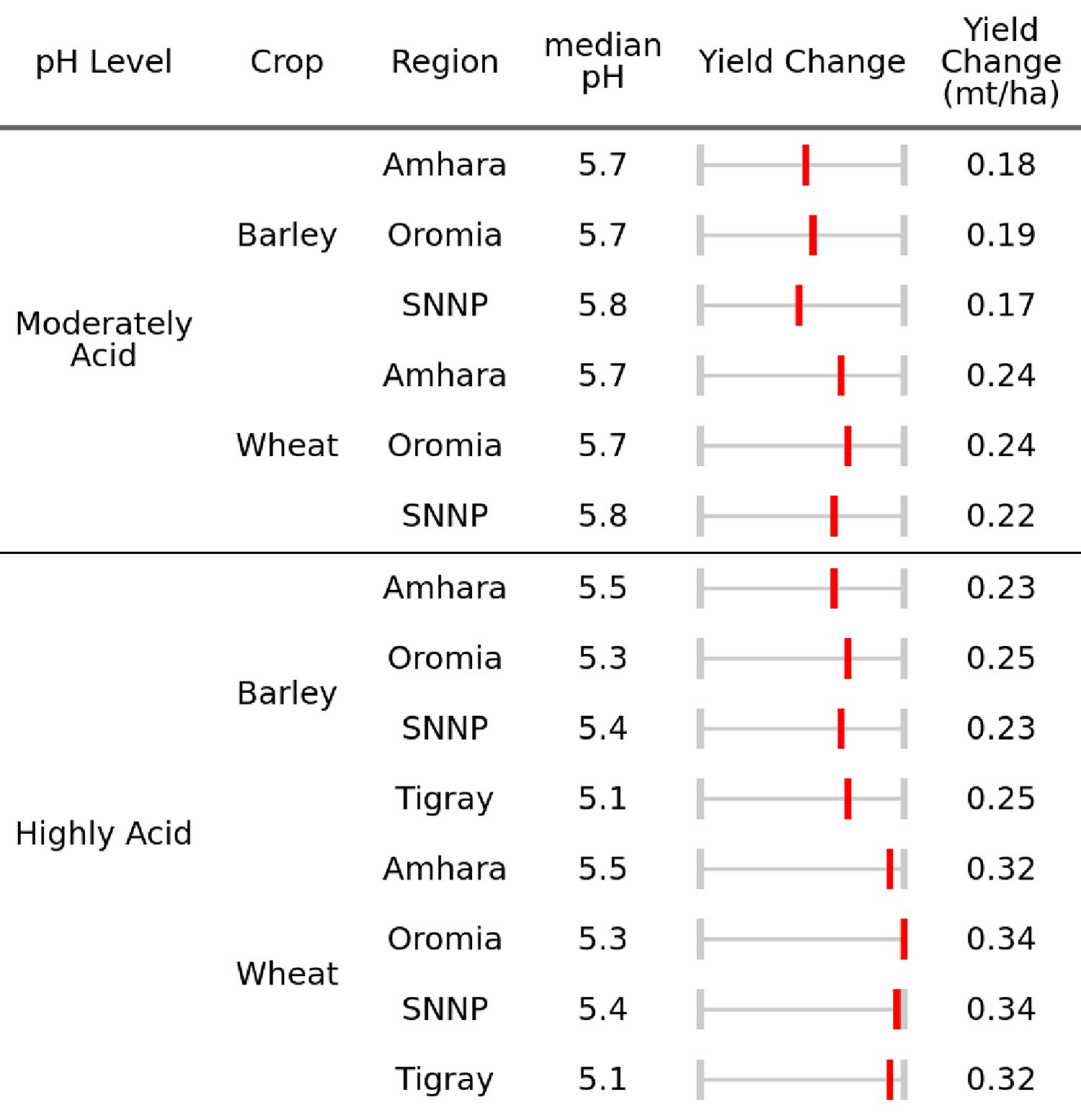
Median yield impact of increasing pH from actual to 6.5 for moderately and highly acidic soils.

The impacts of lime application will vary, following the spatial patterns of soil pH at the sub-kebele level. As such, there is a great deal of variability within and between regions. Careful lime targeting will be required for an effective intervention. While there are “moderately” (5.5 < pH ≤ 6) acidic soils throughout our four regions of study, our AgSS sample in Tigray covers no moderately acidic areas and a relatively small number of highly acidic ones. This reflects the general lack of non-acidic soils in the Tigray regions as seen in Fig 6 below.

To better understand the spatial variability in yields obtainable by increasing the current pH to 6.5, we use the polynomial estimates from models 1 and 3 and plot the results in Fig 6.

Differences in Fig 6 are strongly influenced by crop type and how pH varies by region. In highly acidic soils (pH ≤ 5.5), substantial yield gains are likely obtainable by properly adjusting the pH balance. The median gains for wheat and barley are 0.33 and 0.24 (mt/ha), respectively. This uniform yield response across regions can be explained by the overriding influence of a pH significantly at or below a pH of 5.5. In moderately acidic soils (5.5 < pH ≤ 6) these benefits attenuate and vary region-by-region according to their differences in mean pH.

### d) Economic costs comparison of lime and fertilizer

In this section, we compare the present value of applications of lime and fertilizer required to obtain the same yield increase (0.3 and 0.2 mt/ha for wheat and barley from Table 4) for both interventions for the simplified case (moving pH from 5.5 to 6.5 and holding the delivered lime price at is mean).

For soils with a pH of 5.5, the average present value of fertilizer use for wheat is negative and 2.6 times that of a single application of lime (Table 5). Implying for wheat planted in soils with a pH of 5.5, fertilizers are 260% more expensive than lime for a 0.3 mt/ha increase in yield. Meanwhile the present value of a comparable fertilizer application for barley is 1.1 times that of lime, or in other words, for barley, fertilizer is 10% more expensive than lime. As such, lime can likely provide substantial savings particularly for wheat but also barley farmers.

**Table 5:** Average present value of lime and fertilizer applications required to obtain yield increase equivalent to moving pH from 5.5 to 6.5.

Crop	Treatment	Present Value	Yield Increase	x Cost of Lime
		(ETB)		
All	Lime	-5,266.2	-	1
Wheat	Fertilizer	-13,793.0	0.3	2.6
Barley	Fertilizer	-5,812.6	0.2	1.1

Following the approach outlined in the methods section we now calculate a spatially explicit cost comparison for both lime and fertilizer. Below, spatially explicit estimates of fertilizer and lime costs are aggregated by acidity class, region, and crop, reflecting the median regional soil properties and input costs.

**Fig 7:**
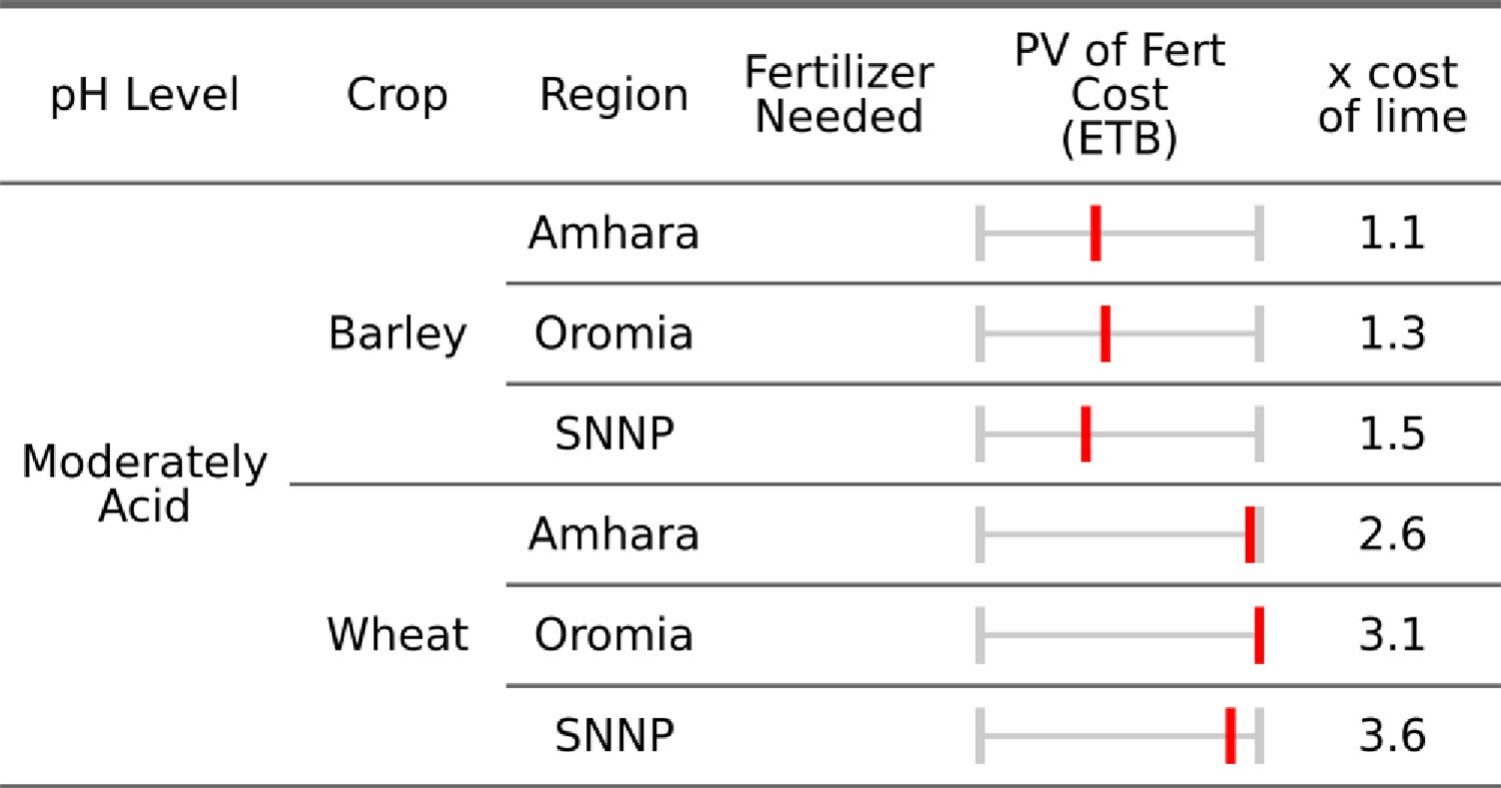
Median regional fertilizer requirements to match lime application (minimum pH of 6.5) for moderately acidic soils.

From an economic standpoint Fig 7 demonstrates that for even moderately acidic soils the annual application of fertilizer is more costly than an application of lime in all regions and both crops, ranging from 110–150% more. Tigray is excluded because no moderate pH sub-kebeles were in the AgSS sample. In the case of wheat, the application of lime is significantly cheaper than the use of fertilizer. Overall, these estimates imply a cost savings of, excluding Tigray, 6,578 ETB and 1,037 ETB per hectare of limed land, for wheat and barley respectively. Even in moderately acidic soils these values are a large portion of average one-year agricultural household income, which were 8,176ETB or $370 in 2016 [48]. As such, the cost savings of lime application to wheat was 80% of annual agricultural income, and 13% for barley spread over a five-year period. These benefits however are unevenly distributed, following spatial patterns of soils and transportation costs across regions.

**Fig 8:**
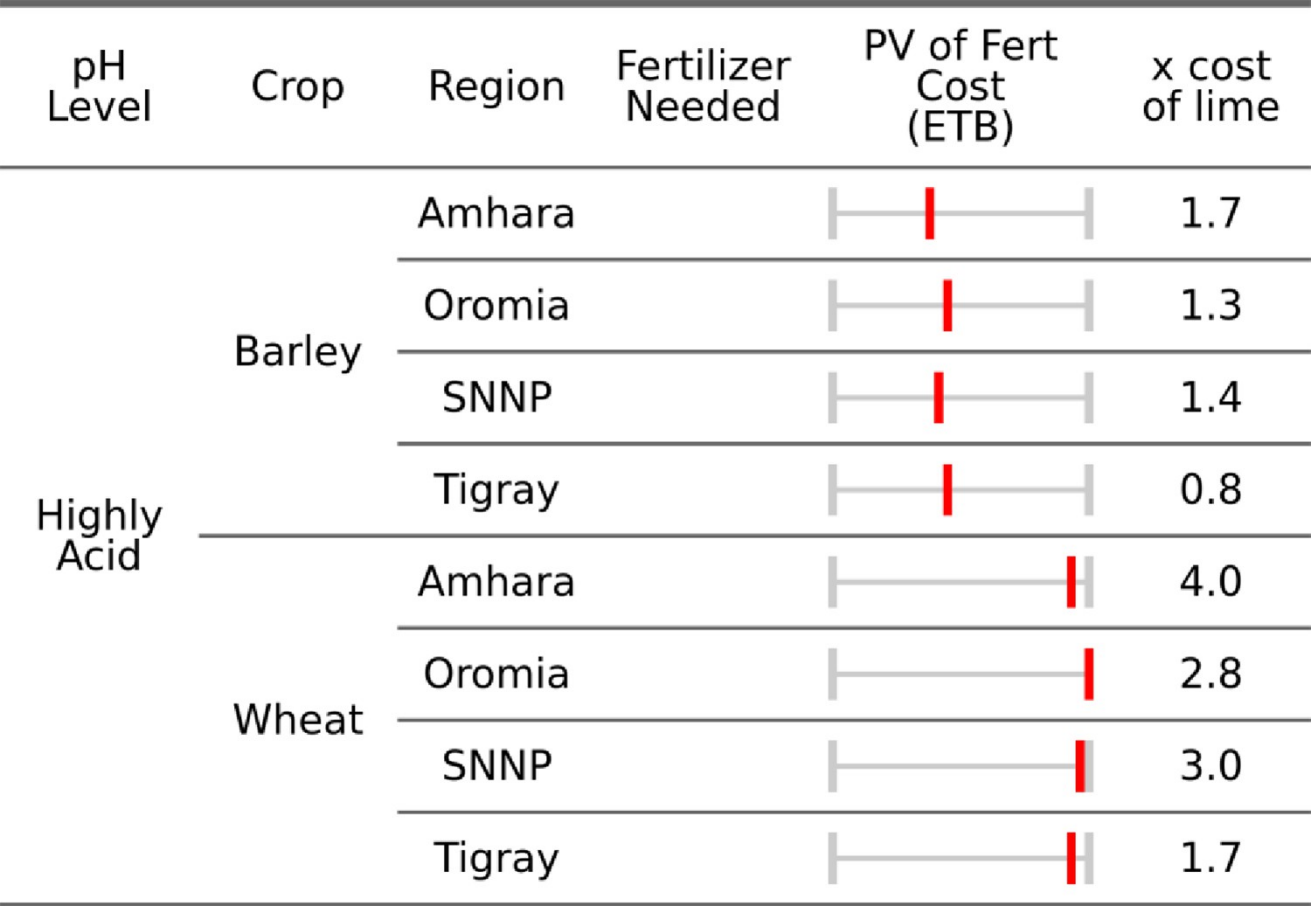
Median regional fertilizer requirements to match lime application (minimum pH of 6.5) for highly acidic soils.

For highly acidic soils, presented in Fig 8, the economic gains of lime applications become clear, especially for wheat. Across all growing regions, the economic benefit of lime application for wheat in highly acidic soils is 2.9 times that of fertilizer applications but reaches as high as four times. For wheat and barley respectively, this implies a cost savings of, excluding Tigray, 9,901 and 1,930 ETB per hectare of limed land, or 121% and 24% of annual household agricultural income spread over a five-year period.

**Fig 9:**
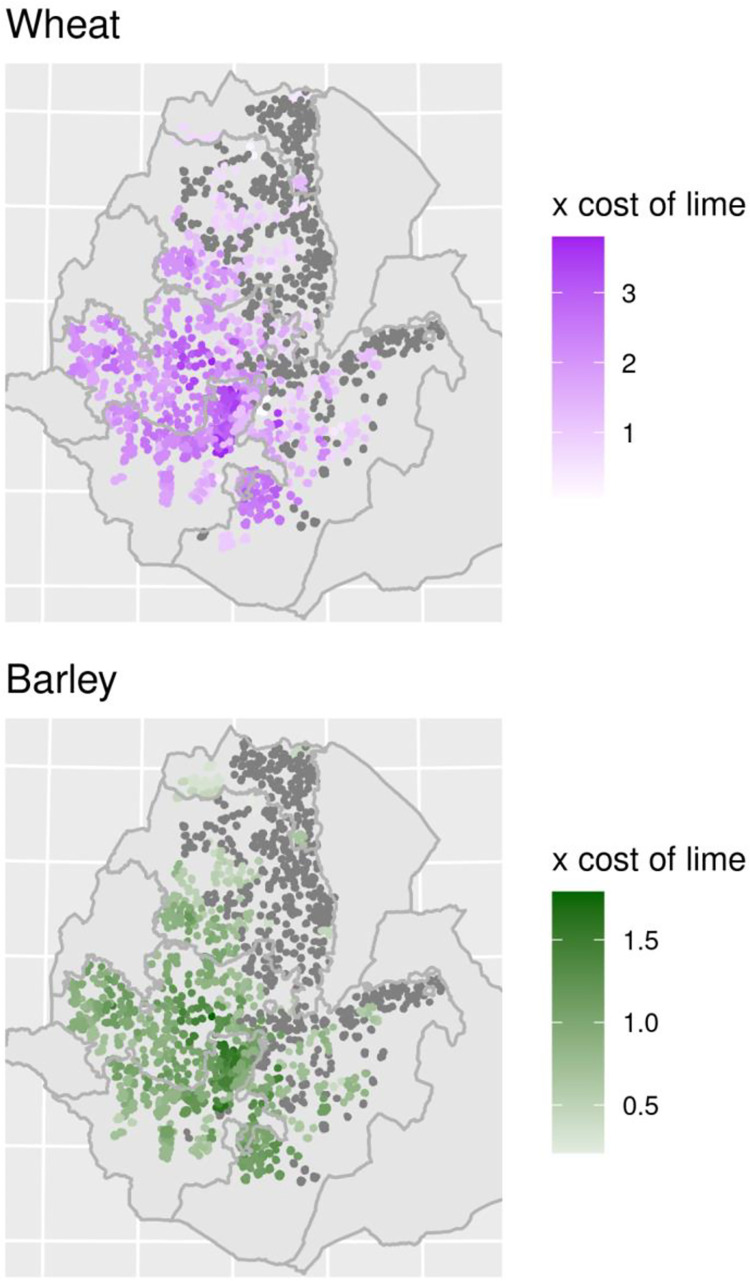
Present value ratios of fertilizer relative to lime application for wheat and barley. Contains information from OpenStreetMap and OpenStreetMap Foundation, which is made available under the Open Database License.

Fig 9 maps the present value ratios of fertilizer and lime interventions across the regions at the sub-kebele level. As in the two previous tables, a value of three for “x costs of lime” indicates that annual fertilizer applications are three times more expensive than a single lime application over a five period. Areas in grey indicate that fertilizer is likely more effective, largely due to the fact that soils are not acidic. This implies that the introduction of lime can provide substantial yield gains while being more cost effective than applications of additional fertilizer. According to our data, the greatest benefits for application of lime are located in northern SNNP and western areas of Amhara and Oromia, largely following the patterns to highly acidic soils. We also present a web map version of the same data at our website.

An important caveat to this research is that the data is aggregated at the sub-kebele level, and our model largely exploits spatial variability in yields and soil properties rather than through controlled trials or farmer experience. As such, the results should be viewed as indicative rather than specific to individual farm plots. At the plot-level, given the results of small-scale trials and these results, we believe that productivity enhancements from lime would likely exhibit both greater variation and higher average yields. However, farm level trials would be needed to confirm this hypothesis.

The evidence provided here outlines the potential economic benefits of Ethiopia implementing a similar strategy to Brazil’s, whereby lime was applied acid soil reclamation in their Cerrado region [49, 50]. The Brazilian initiative brought together government resources, the national research institute, and international technical and financial aid to carry out an intensive rehabilitation initiative that resulted in the reclamation of 60 million ha of farmland and created a global production source for wheat. We believe that the Brazilian experience provides an important international template in the possibilities of acid soils management and envision a similar strategy that will have substantially positive impacts for Ethiopia. In addition, given several other African countries growing interest in the impacts of soil acidity, this prototype could serve as a foundation for other countries. The goal is enhanced production by directly addressing the economics of soil health for improving farmer’s livelihoods in Ethiopia and other African countries.

Finally, the benefits of lime application extend beyond simple yield gains and are worth emphasizing. For example, the benefits of lime application can include: increased Ca Mg and P supply, reduced mineral toxicity, stimulation of microbial activity, avoiding induced acidification through fertilizer application, and more efficient uptake of N and availability, among others. Additionally, although not explicitly modeled here, it is likely that the application of lime could also allow farmers to reclaim abandoned farmland, thereby substantially increasing total production in highly acidic areas. Therefore, we recommend targeted lime application as part of a greater policy of integrated soil fertility management that includes fertilizer as well as other potential inputs and farm management strategies.

## 4. Conclusion

Acid soils are a significant and growing problem for agricultural productivity in Ethiopia. Restoring soil pH to optimal ranges for acid intolerant crops, like wheat and barley, could provide a significant boost to productivity at a cost lower than fertilizer. Our results indicate that moderately and highly acidic soils significantly undermine yields for major cereal crops and estimate the impact of moving pH from 5.5 to 6.5, through a lime remediation strategy, could increase yields by 22% and 19% for wheat and barley, respectively. Moreover, using spatially explicit soil and input price data we find evidence on a region-by-region basis for the economic viability of soil remediation through the application of lime, especially in highly acidic areas. At the regional level, taking into account transportation costs, the benefits of lime applications for wheat are as much as 4 times that of fertilizer, or 2.9 across all major growing regions. In highly acidic soils this implies a cost savings of, excluding the region of Tigray, 9,901 ETB and 1,930 ETB per hectare of limed land, for wheat and barley respectively. Moreover these savings are a substantial proportion of the one-year agricultural income per hectare, (121% and 24% for wheat and barley).

Taken as a whole, there is substantial evidence that soil remediation can increase yields and economic outcomes for farmers across its acidic regions. These findings do not argue for an “either or decision,” choosing between lime and fertilizer, but instead demonstrate that lime should be considered an important and cost-effective intervention in Ethiopia. Extensive research indicates that balanced pH is an essential prerequisite to optimal crop yields, especially for crops such as wheat and barley [28]. Therefore, we recommend targeted lime application as part of a greater policy of integrated soil fertility management that includes fertilizer as well as other potential inputs and farm management strategies.

## Supporting information

S1 File (DOCX)
