# Supplementary material for: Estimating acid soil effects on selected cereal crop productivities in Ethiopia: Comparing economic cost-effectiveness of lime and fertilizer applications
Source: PLoS One. 2023 Jan 12;18(1):e0280230. doi: 10.1371/journal.pone.0280230 (PMC9836257; doi:10.1371/journal.pone.0280230)
Supplement: S1 File — (DOCX) [file pone.0280230.s001.docx]

**6. Supplemental Material**

# Appendix A – Data

## AgSS

The AgSS is a large-scale survey tasked with measuring agricultural production in Ethiopia at the zonal level. Each year the AgSS interviews approximately 45,000 farmers on a range of farm management questions covering some basic demographics of the household as well as a range of questions concerning planting, harvesting and selling at the plot level. Typically, about 20 farm households are randomly sampled from village-level areas of approximately 200 households (the sub-kebele level). From this sampling frame, a random selection of about 2,200 sub-kebeles are chosen as a representative sub-sample for zonal level agricultural production. Population weights are then applied to project agricultural production at the zonal level. For this study, we construct longitudinal data over the six crop seasons and are able to maintain 75% of all households over the 2010-2016 period at the sub-kebele level.

In 2017, the Central Statistics Agency employed a significantly different research design for collecting AgSS data. To maintain the integrity of the longitudinal data set, data beyond 2016 were not used. This also aligns with the EthioSIS soil data collection period described below.

While this study collected data on the five principal Ethiopian field crops (teff, wheat, maize, barley, sorghum), we focus here on wheat, and barley because of their importance in Ethiopia’s food system, as well as their sensitivity to soil acidity. The principal unit of analysis is the sub-kebele and all relevant CSA data is aggregated to this level. In our analysis, we amalgamate the production of all 20 households in a sub-kebele as a single representative farmer. This was done for a variety of reasons including the fragmented plot farming system common in Ethiopia as well as CSA data collection methodology. More specifically, CSA data collection methodology relies on crop cuts to estimate productivity at the local level. Depending on the actual number of farmers growing the specific crop, CSA collects up to five different individual farmer crop cuts, averages the yields, and projects this figure onto all plot areas for that crop in the sub-kebele.

## EthioSIS

EthioSIS is a national soil survey and mapping exercise conducted by the Government of Ethiopia, and competed in 2019 [29]. The objective was to create both a national and woreda-level mapping grid that identifies soil fertility characteristics of agricultural lands for the purpose of assisting in fertilizer advisory service to farmers. The project combined field observation, soil data collection and remote sensing into a comprehensive soil inventory. Over 100,000 soil samples were collected using pre-defined, systematically stratified sampling points of topsoil (0 -20 cm) vs (0-50cm), to determine soil fertility at a 250 m resolution. The specific process included using a sampling frame based on geo-referenced gridded locations, lab analysis of samples and predictive models of soil properties as a function of spatial covariates.

## TerraClimate

TerraClimate provides monthly estimates of surface water balance at 1/24th of a degree (~4km) spatial resolution. Measures of water balance are particularly relevant to this study because it provides an estimate of water available to plants by looking at the balance between water gained from precipitation and runoff, and that lost to evaporative demand. The amount of water moving through a system is driven by two forces: (1) the supply of water through rainfall and (2) the demand for water, driven primarily by energy from the sun, which allows for evaporation and movement of water through plants but is also affected by wind, vapor pressure, and soil properties. Here we use a measure called the Palmer Drought Severity Index which is derived as a measure of the balance between available water due precipitation and potential evapotranspiration [30].

## PSNP & AGP

The AGP intervention is designed to increase productivity and marketed output within high potential agricultural areas [51]. Ethiopia’s PSNP is an internationally recognized safety net program that provides a variety of social programs to assist poorer households. There are approximately 200 woredas designated as PSNP woredas and the program was initiated in 2005 [52]. The AGP intervention is a large-scale (83 initial woredas) project, funded by the World Bank, designed to increase productivity and marketization within high potential agricultural areas. Intervention woredas were obtained from the Ethiopia Strategy and Support Program II’s baseline report [51]. Ethiopia’s PSNP is an internationally recognized safety net program that provides a variety of social programs to assist poorer households. There are approximately 200 woredas designated as PSNP woredas and the program was initiated in 2005 [52].

## Intermediate Products

To better measure needed lime requirements the soils “buffer capacity” is projected as a function of sub-kebele values of CEC as well as base saturation estimates values determined by a pH-base saturation model [9]. Typically, CEC is used for pH measurements above 6.0 and exchange acidity (EA) is used for lower pH levels. Because of EthioSIS data limitations, we use CEC for all pH levels. Tillage depth (TD) is assumed to be about 15 cm (5.9 in) for traditional oxen plowing in Ethiopia [53]. Calcium carbonite equivalent is assumed to be 100% (eg. Calcitic limestone) with 100% fineness (F) for quick absorption into the soil. Finally, we index the equation to approximately 3 mt/ha for a 1 unit increase in pH (5.5 to 6.5) to conform with Ethiopian soil scientists’ expert opinion on general national liming rate requirements. Sub-kebele estimates of the lime required to obtain a pH of 6.5 shown in Figure A1 below.

Figure A1: Estimate of average lime required per hectare to amend soil pH at multiple pH levels


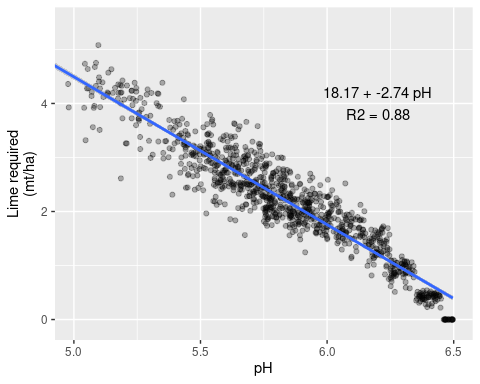


Estimates of delivered input distributors lime costs are shown in a Figure below.

**Figure A2: Map of estimated input distributors prices of lime**

Contains information from OpenStreetMap and OpenStreetMap Foundation, which is made available under the Open Database License.


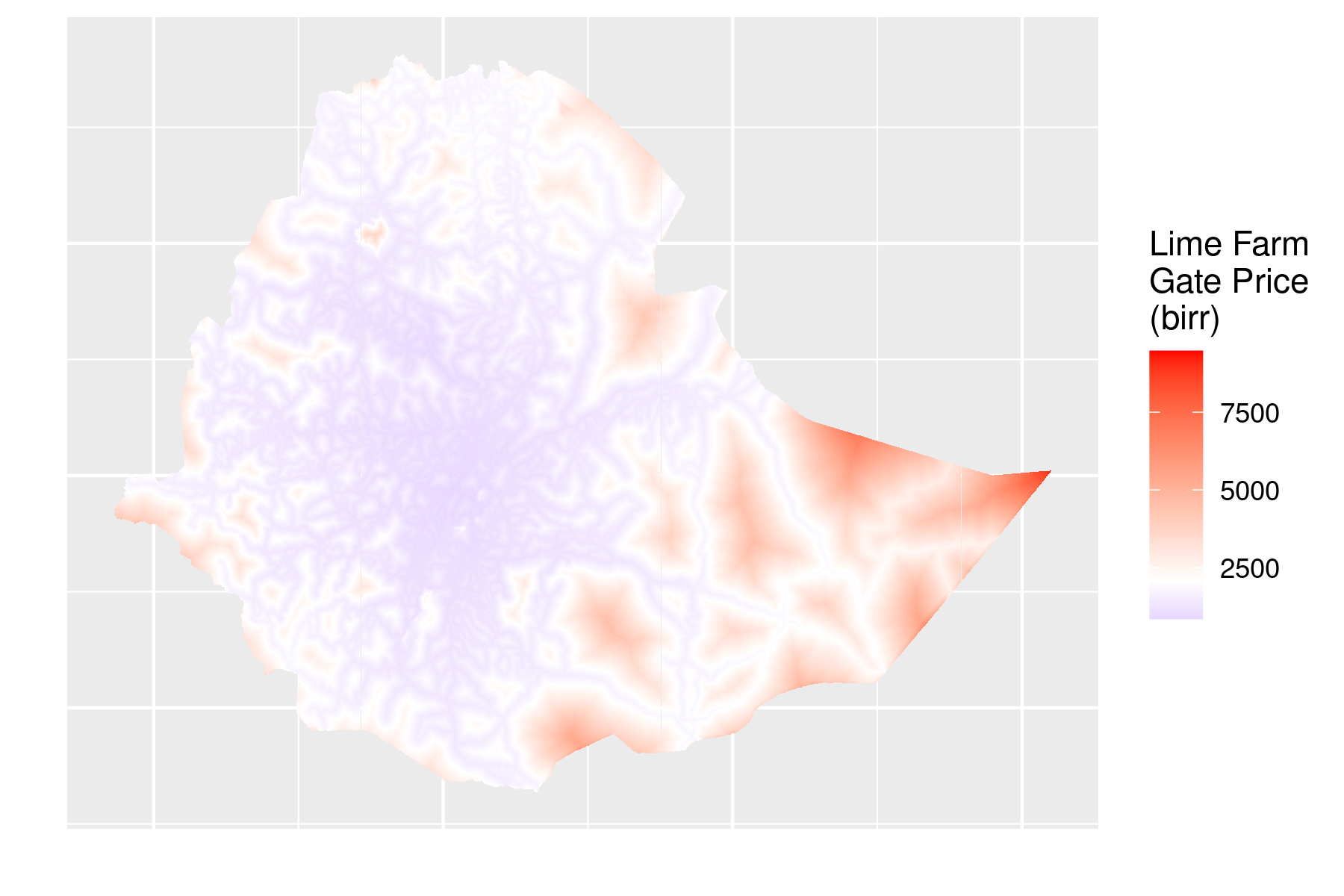


# Appendix B - Results

## Yield gains from soil remediation

We can also look at the spatial distribution of these gains in productivity from the application of lime. Below we can see the impact of increasing pH to a minimum of 6.5 for both barley and wheat.

**Figure B1: Change in wheat yields obtained by increasing pH to a minimum of 6.5**

Contains information from OpenStreetMap and OpenStreetMap Foundation, which is made available under the Open Database License.


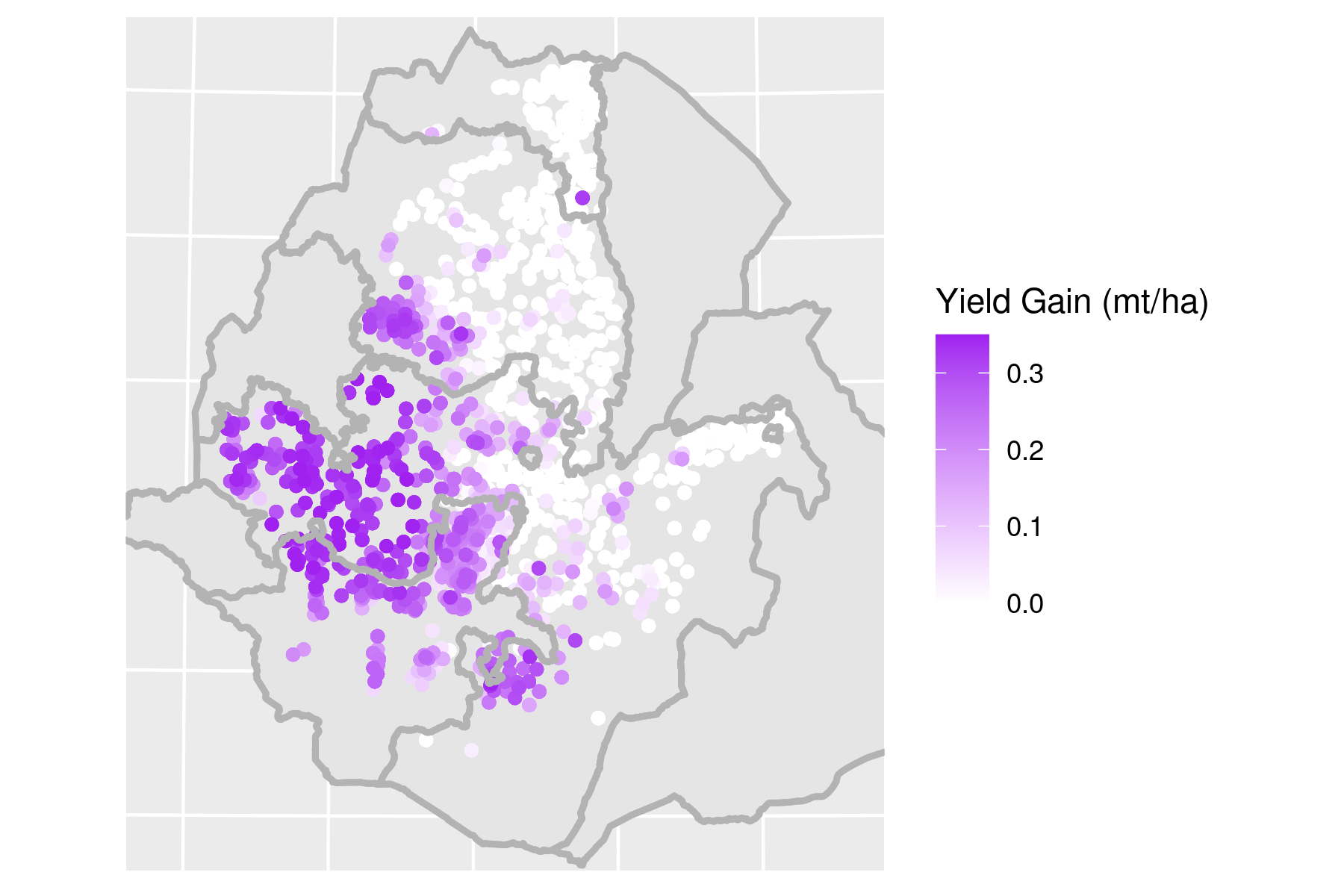


**Figure B2: Change in barley yields obtained by increasing pH to a minimum of 6.5**

Contains information from OpenStreetMap and OpenStreetMap Foundation, which is made available under the Open Database License.


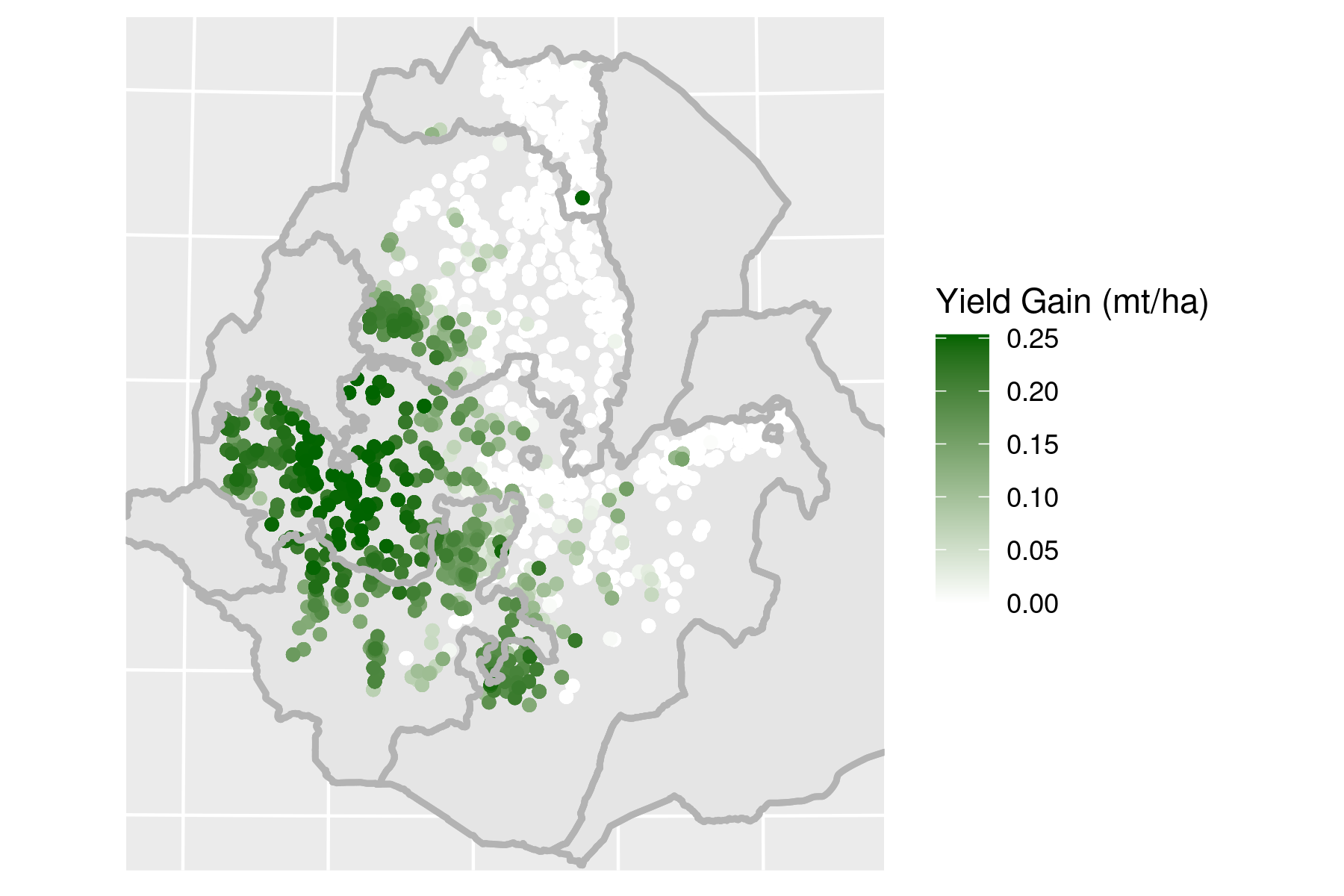


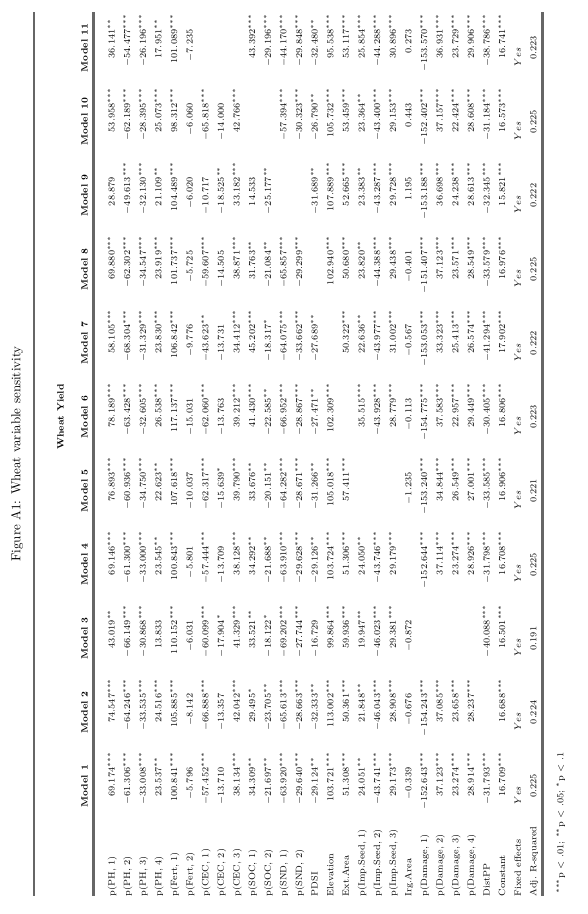


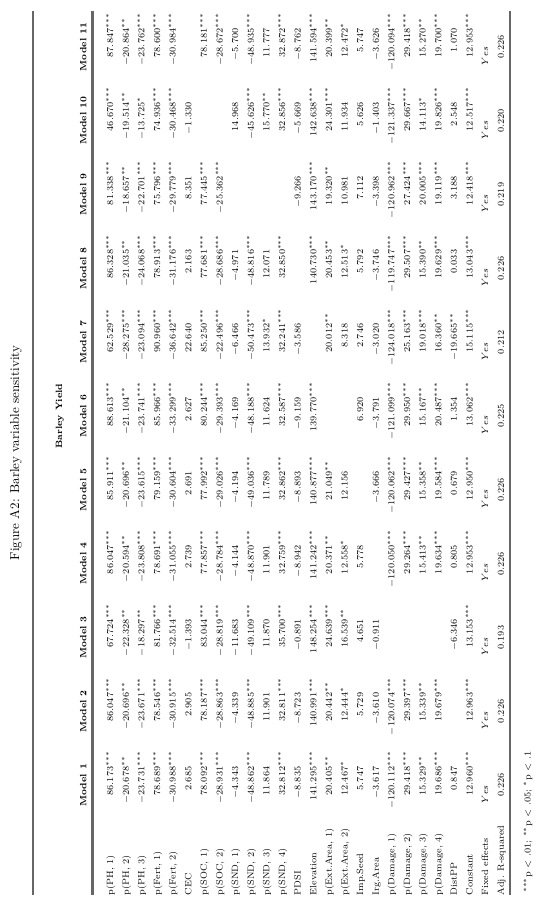


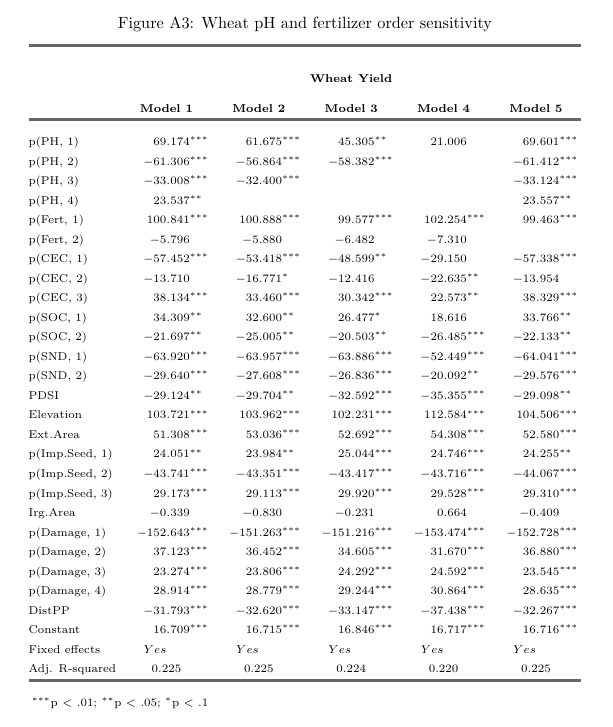


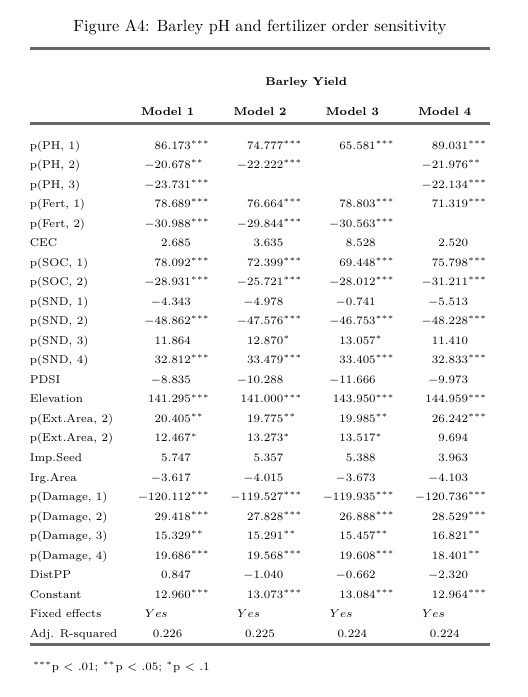


# References

9. Quirine Ketterings JB Kristen Stockin, Miller J. Lime guidelines for field crops; agronomy fact sheet series fact sheet 6. Department of Crop and Soil Sciences Cornell University, Ithaca NY. 2006.

29. EthioSIS. Ethiopian soil information system (EthioSIS). 2015. Available: <http://www.ata.gov.et/highlighted-deliverables/ethiopian-soil-information-system-ethiosis>

30. Abatzoglou JT, Barbero R, Wolf JW, Holden ZA. Tracking interannual streamflow variability with drought indices in the US pacific northwest. Journal of Hydrometeorology. 2014;15: 1900–1912.

51. Berhane D G. Agricultural growth program (AGP) of Ethiopia–baseline report 2011. ESSP/EDRI Report International Food Policy Research Institute, Addis Ababa. 2013.

52. Gilligan D, Hoddinott J, Taffesse AS, Dejene S, Tefera N, Yohannes Y. Ethiopia food security program: Report on 2006 baseline survey. International Food Policy Research Institute, Washington, DC Photocopy. 2007.

53. Abidela Hussein M, Muche H, Schmitter P, Nakawuka P, Tilahun SA, Langan S, et al. Deep tillage improves degraded soils in the (sub) humid Ethiopian highlands. Land. 2019;8: 159.
